# Development of seropositivity to SARS-CoV-2 over the course of the COVID-19 pandemic in adolescents in a longitudinal cohort study in Cebu, Philippines

**DOI:** 10.1371/journal.pgph.0005961

**Published:** 2026-02-26

**Authors:** Jedas Veronica Daag, Gianne Lariz Magsakay, Silvia Blanco Rivera, Ma. Gladys Nicole Daque, Irish Lobitana, Marla Angela Lariana, Kiarah Louise Florendo, Maria Vinna Crisostomo, Ana Coello Escoto, Lakshmanane Premkumar, Rosemary Aogo, Jacqueline Deen, Leah Katzelnick, Michelle Ylade

**Affiliations:** 1 Institute of Child Health and Human Development, National Institutes of Health, University of the Philippines-Manila, City of Manila, Philippines; 2 Viral Epidemiology and Immunity Unit, Laboratory of Infectious Diseases, National Institute of Allergy and Infectious Diseases, National Institutes of Health, Bethesda, Maryland, United States of America; 3 Department of Microbiology and Immunology, University of North Carolina School of Medicine, Chapel Hill, North Carolina, United States of America; Mahidol University, THAILAND

## Abstract

COVID-19 was first reported in the Philippines in January 2020 and the country experienced several COVID-19 waves. Little is known on the seroprevalence of SARS-CoV-2 among adolescents in a developing country such as the Philippines. In this study, we incorporated testing for acute COVID-19 and SARS-CoV-2 seropositivity in an ongoing dengue study of 9- to 14-year-old participants enrolled in 2017. Acute febrile episodes were detected through active surveillance and a nasopharyngeal swab was obtained for SARS-CoV-2 RT-PCR. Before COVID-19 vaccination was offered, we tested a subset of sera obtained in 2021 for SARS-CoV-2 receptor binding domain (RBD) antibodies. Development of SARS-CoV-2 seropositivity acquired through natural infection and vaccination was conducted by measuring antibodies pre- (2018–2019), early- (2019–2020), and late-pandemic (2022–2023) in those with matched samples. From July 2021-October 2022, we recorded 29 acute febrile illness episodes in the cohort and 17.0% were SARS-CoV-2 RT-PCR positive. In the subset of 499 samples tested for SARS-CoV-2 RBD antibodies in 2021, 30.3% were seropositive. Among these, 4.0% were seropositive in 2018–2019, and 11.0% had seroconverted by 2020. Interestingly, 74.0% experienced antibody waning between 2020 and 2021 sampling. By 2022, 345/499 children received full COVID-19 vaccination and 13/499 was partially vaccinated, with 96.6% SARS-CoV-2 seropositive by late-pandemic sample in 2022–2023. Vaccination and site were predictors of SARS-CoV-2 RBD antibodies. These findings suggest both variation in local transmission over time as well as association of vaccination with the development of SARS-CoV-2 immunity in adolescents in the Philippines.

## Introduction

In December 2019, severe acute respiratory syndrome coronavirus 2 (SARS-CoV-2), which causes coronavirus disease 2019 (COVID-19), emerged in Wuhan, China and rapidly spread around the world. By early 2020, sustained community transmission had been documented across multiple regions, prompting the implementation of large-scale non-pharmaceutical interventions and the accelerated development of vaccines [1].

In the Philippines, the first confirmed COVID-19 case was reported on 30 January 2020, involving a 38-year-old female from Wuhan testing positive for the virus [2]. The Philippine government implemented a multi-sectoral response including community quarantine and lockdowns, expansion of its testing service, financing of the isolation and hospitalization of cases and economic assistance of low-income families. Despite these control measures, the country experienced several COVID-19 waves with over four million cases reported to date [3]. The first wave peaked in mid-2020, the second and third (highest) waves in 2021, the fourth wave in early 2022 and followed by smaller waves thereafter [3].

Early surveillance in the Philippines relied on case-based reporting and facility-based testing, which disproportionately captured adult and symptomatic infections. As in many settings, infections among children and adolescents were likely under-ascertained due to milder clinical presentations, lower testing uptake and barriers to diagnostic access [4,5]. Consistent with this, cross-sectional studies have shown SARS-CoV-2 seropositivity of over 40% in Manila prior to the roll-out of COVID-19 vaccines [6,7]. However, population-based data among children and adolescents remained scarce. A comprehensive review conducted by Naeimi et al. showed that pediatric SARS-CoV-2 seroprevalence varied across pandemic waves, age groups, ethnicity, income levels and World Health Organization (WHO) regions [8].

Nationwide mass vaccination against SARS-CoV-2 was started in adults in March 2021 and was extended to adolescents in November 2021 [9]. Of the estimated population of 112,892,781 in the Philippines in 2023 [10], nearly 80 million have been fully vaccinated against SARS-CoV-2 [11]. While vaccination substantially altered population immunity, the contribution of natural infection and vaccination to immunity among adolescents, and how these evolved across successive pandemic phases, remain uncharacterized, especially in low- and middle-income settings.

To address these gaps, we incorporated a COVID-19 component in an ongoing prospective cohort study of adolescents originally established for dengue surveillance. This design enabled the use of archived pre-pandemic samples and longitudinal follow-up to assess the evolution of SARS-CoV-2 seropositivity among adolescents across pre-, early-, mid-, and late-pandemic periods in a low- and middle-income setting.

## Materials and methods

### Ethics statement

This study was reviewed and approved by the University of the Philippines – Manila Research Ethics Board (UPM-REB, Protocol Code: 2020-773-01). A parent or legal guardian of the participants provided written informed consent. Verbal assent was obtained from the participants and documented.

The study was performed following the Strengthening the Reporting of Observational Studies in Epidemiology (STROBE) guidelines [12]. The participant pool were from a larger, longitudinal dengue study (clinicaltrials.gov number: NCT03465254). Stored serum samples collected since 2018 until 2023 from the dengue study were used after a broad consent was obtained and were accessed on 01 August 2021. Participants from the dengue study who reported an acute febrile illness from 01 July 2021–31 October 2022 were invited to participate in a prospective surveillance for SARS-CoV-2 infection.

### Study population and design

**Fig 1:**
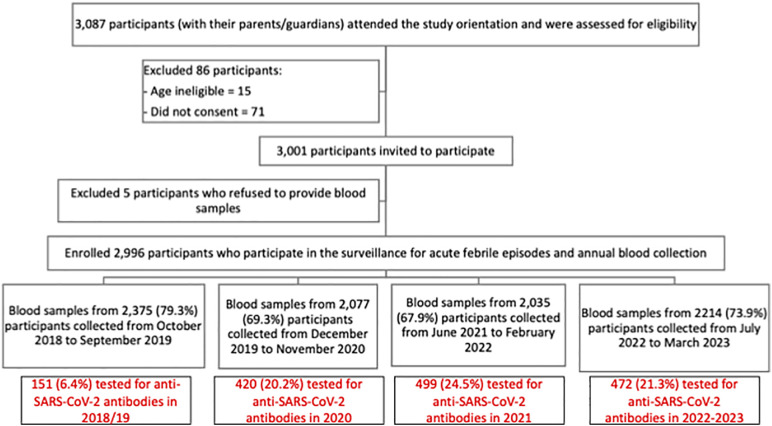
Flow chart of study participant enrolment and annual blood sample collection for the dengue cohort and samples tested for SARS-CoV-2 antibodies.

From 2 May to 2 June 2017, 3,087 participants (with their parents or guardians) attended the study orientation and were assessed for eligibility. We invited 3,001 into the dengue study and enrolled 2,996. The study was conducted in Bogo and Balamban, both semi-urban areas in Cebu province. The sera were collected as part of a five-year on-going cohort study (ClinicalTrials.gov, NCT03465254) [13]. In 2017, healthy participants who were 9–14 years of age were enrolled and followed for acute febrile illness. Demographic and socio-behavioral data were recorded using a standard case report form. From each participant, approximately 5 ml of blood were collected in anticoagulant-free vacutainer tubes at baseline in 2017. We obtained an annual blood sample from the participants four times from 2018 to 2023 (Fig 1). The sera were processed, aliquoted and stored in -80°C prior to testing. We randomly selected a subset of serum samples (see sample size calculation below) collected from June to October 2021 during the third wave and before COVID-19 vaccination was offered to adolescents (timepoint 3 sampling in the dengue cohort) for SARS-CoV-2 serologic testing. The selection was done using computer-generated random numbers. We also tested all available paired samples collected at the start of the pandemic (timepoint 2, December 2019 to November 2020) and later in the pandemic (timepoint 4, July 2022 to March 2023). For all children with positive SARS-CoV-2 antibodies in year 2 or year 3, samples collected before the pandemic (timepoint 1, October 2018 to September 2019) were tested to confirm negative status prior to the pandemic.

### Vaccine status

Vaccine product and administration dates was determined by referencing vaccine cards. All but one of the timepoint 3 samples were collected before COVID-19 vaccination was initiated in the Philippines in adolescents. One participant was vaccinated 4 days before timepoint 3 sample collection, and this occurred because the participant was 18 and thus an adult prior to initiation of adolescent vaccination campaigns. All timepoint 4 samples were collected after the 2nd vaccine dose.

### Confirmation of acute febrile illnesses as COVID-19 cases

In the dengue cohort study, participants were actively monitored through monthly contact for occurrence of an acute febrile illness. Acute febrile illness was defined as fever occurring <5 consecutive days or presence of illness including cough, colds, abdominal pain, body malaise or generally feeling unwell. Once an acute febrile illness report was received, participants came to the study site where another consent was obtained for participation in the SARS-CoV-2 study. Upon obtaining consent, COVID-19-related clinical information were recorded, and a nasopharyngeal sample was collected and sent to an accredited local laboratory for RT-PCR. Acute COVID-19 cases were referred to a health facility for management and case-finding according to national guidelines.

### Assessment of antibodies to the receptor binding domain of SARS-CoV-2

The receptor binding domain (RBD) of the spike protein is highly specific to each coronavirus and is an important target of neutralizing antibodies [14,15]. Stored serum samples were tested for antibodies to the spike RBD of SARS-CoV-2, according to a previously published ELISA procedure, after heat-inactivated at 56°C for 30 min to reduce the risk from any possible residual virus [16]. Briefly, 50 microliters of spike RBD antigen at 4 microg/ml in tris-buffered saline (TBS) (pH 7.4) was coated in a 96-well high-binding microtiter plate (Greiner Bio-One, catalog no. 655061) for 1 hour at 37°C. Then, the coating solution was discarded, and the plate was blocked with 100 microliters of blocking solution (3% milk in TBS containing 0.05% Tween 20) for 1 hour at 37°C. The blocking solution was removed, and 50 microliters of serum sample at 1:40 or indicated dilutions in blocking buffer was added for 1 hour at 37°C. The plate was washed with the wash buffer (TBS containing 0.2% Tween 20), and 50 microliters of alkaline phosphatase-conjugated secondary goat anti-human secondary antibody at 1:2500 dilution was added for 1 hour at 37°C. For measuring total immunoglobulin (Ig), a mixture of anti-IgG (Sigma, catalog no. A9544), anti-IgA (Abcam, catalog no. AB97212), and anti-IgM (Sigma, catalog no. A3437) were added together. The plate was washed, and 50 microliters of p-nitrophenyl phosphate substrate (SIGMAFAST, catalog no. N2770) was added to the plate and absorbance was measured at 405 nm using a plate reader (Biotek Epoch, model no. 3296573). Positivity for ELISA optical density (OD) values were determined relative to a negative control and a previously established threshold for positivity [16]. We interpreted the results as follows: an OD result of less than 0.3 was considered as negative (no evidence of past SARS-CoV-2 infection) and ≥0.3 was considered as positive (presence of detectable antibodies indicating a past or recent SARS-CoV-2 infection) [16].

### Sample size calculation and data analysis

The primary endpoint of the study was the SARS-CoV-2 seroprevalence measured at timepoint 3. The sample size required to estimate the seroprevalence in a cohort of 2,996 participants is 228 or 341 assuming a true seroprevalence of 20% or 50%, respectively, a desired precision of the estimate of 0.05 and confidence level of 0.95 [17]. To have a sufficient number for the assessment of persisting anti-SARS-CoV-2 after natural infection, we used a final sample size of 499 after adding 158 (30%) for potential loss to follow up.

Analysis was done using Stata 17.0 (StataCorp, 2021) and R Foundation for Statistical Computing (R version 4.2.1). We compared demographic and socio-economic variables collected as part of our dengue study between participants who were SARS-CoV-2 seropositive and seronegative by paired t-test for continuous variables and Pearson’s χ^2^ test for categorical variables. A P ≤ 0.05 was considered as statistically significant. Sample collection dates were visualized as histograms. Changes in ELISA OD values were visualized using boxplots showing medians and inter-quartile ranges with whiskers extending to cover 95% of the data, with individual samples shown as dots, and paired samples connected by lines. Significant changes in ELISA OD values were evaluated using paired t-tests with Bonferroni correction. The effect of age, sex, site, and vaccination status on the magnitude of anti-SARS-CoV-2 RBD OD values in 2022/2023 was estimated using linear regression.

## Results

### Acute cases

**Fig 2:**
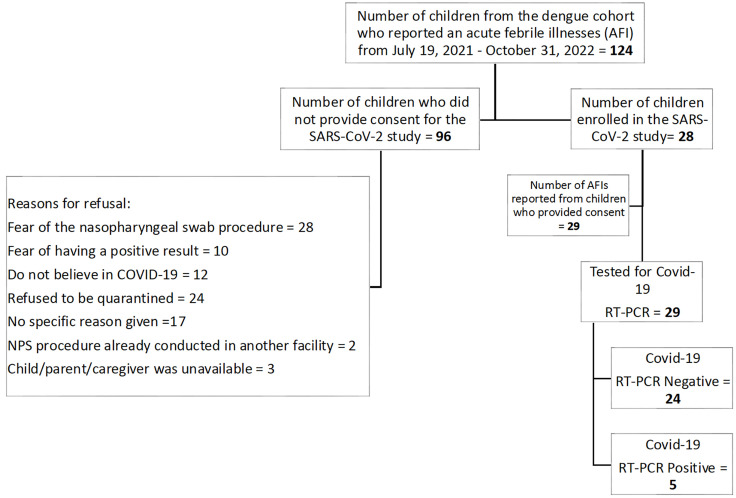
Flow chart of study participant enrolment for the SARS-CoV-2 subcohort. * One participant experienced two febrile episodes during the observational period.

From July 2021 to October 2022, 134 acute febrile episodes were reported in the dengue cohort, of which 29 episodes from 28 participants were included in the SARS-CoV-2 study. Participants enrolled in the SARS-CoV-2 study were older compared to participants in the dengue cohort, but no other significant differences were observed (Table 1). Out of 29 acute febrile episodes, 5 (17%) were SARS-CoV-2 RT-PCR positive (Fig 2). Of the five cases, 1 occurred during the delta wave (July 2021), three during the first Omicron wave (January 2022) and one the following fall (October 2022). All were mild and resolved within 2 days. Only 2 of the 5 were part of the 499 children in the SARS-CoV-2 testing cohort, and both received their 2nd vaccines within one month of their infections in January 2022 (S1 Table).

**Table 1:** Comparison of characteristics between participants in the SARS-CoV-2 study and the dengue study.

		Participants in the SARS-CoV-2 study (n = 28)	Participants in the dengue study (n = 2968)	*p-*value
**Sex**	Male, n (%)	14 (50.00)	1437 (48.43)	0.87
	Female, n (%)	14 (50.00)	1531 (51.57)	
**Age** **(in years)**	Mean (SD) age	15 (1.25)	11 (1.40)	<0.0001
	Median (IQR)	14 (2)	11 (2)	
**Residence**	Bogo, n (%) Balamban, n (%)	14 (50.00) 14 (50.00)	1544 (52.02) 1424 (47.98)	0.83

*We used t-test for continuous variables and ***χ^2^****-square-test for categorical variables*

### Seroprevalence

From June to October 2021, serum samples were collected from 1403/2996 (47%) adolescents in the dengue cohort, of whom a random subset of 499/1403 (36%) was tested for SARS-CoV-2 RBD antibodies. Overall, 151 of 499 participants (30.3%) were seropositive. We compared participants who were SARS-CoV-2 RBD seropositive and seronegative in 2021 on multiple demographic and socio-economic variables that have previously been associated with SARS-CoV-2 transmission in children or adolescents [18]. In this cohort, those who were SARS-CoV-2 positive and negative had similar characteristics (Table 2). The only significant differences were that those with SARS-CoV-2 seropositivity were less likely to have a radio and more likely to have a television.

**Table 2:** Comparison of characteristics between participants who were SARS-CoV-2 RBD seropositive and seronegative in 2021, prior to the nationwide roll-out of COVID-19 vaccination in adolescents.

Characteristics	Participants tested	SARS-CoV-2 seropositive (n, %)	SARS-CoV-2 seronegative (n, %)	p-value
	**499**	**151 (30.3)**	**348 (69.7)**	
**Female**		86 (57.0)	187 (53.7)	0.254
**Male**		65 (43.0)	161 (46.3)	
**Age when blood sample was collected**				
Mean (SD)		14.5 (1.35)	14.6 (1.35)	
Median (range)		14 (13–18)	14 (12–18)	
**Residing in**				
Balamban		83 (55.0)	202 (58.0)	0.26
Bogo		68 (45.0)	146 (42.0)	
**Had one or more** non-dengue **febrile episode from February 2020 to June 2021**		15 (9.9)	37 (10.6)	0.41
**Housing material (n = 495)**				
Wood		75 (49.7)	157 (45.1)	0.21
Cement/concrete		76 (50.3)	187 (53.7)	
**Number of individuals in the household (n = 495)**				
1–4		31 (20.5)	86 (24.7)	0.56
5–8		107 (70.9)	231 (66.4)	
>8		13 (8.6)	27 (7.8)	
**Number of children within the household (n = 495)**				
0–2		86 (57.0)	178 (51.1)	0.56
3–5		60 (39.7)	152 (43.7)	
>5		5 (3.3)	14 (4.0)	
**Household head with > 6 years of schooling (n = 495)**		142 (94.0)	330 (94.8)	1.17
**Has access to electricity**		149 (98.7)	339 (97.4)	0.47
**Household ownership of the following:**				
Radio		52 (34.4)	150 (43.1)	0.02
Television		121 (80.1)	251 (72.1)	0.04
Refrigerator		75 (49.7)	178 (51.1)	0.33
Bicycle		41 (27.2)	98 (28.2)	0.38
Motorcycle		83 (55.0)	203 (58.3)	0.20
Mobile phone		148 (98.0)	337 (96.8)	0.49
Desktop/handheld computer		18 (11.9)	44 (12.6)	0.40
Car		4 (2.6)	12 (3.4)	0.33
**Estimated monthly expenditure (pesos,** ₱)				
Up to 5,000		22 (14.6)	51 (14.7)	0.20
>5,000 up to 10,000		69 (45.7)	184 (52.9)	
>10,000		60 (39.7)	109 (31.3)	

### SARS-CoV-2 seropositivity across pandemic phases

**Fig 3:**
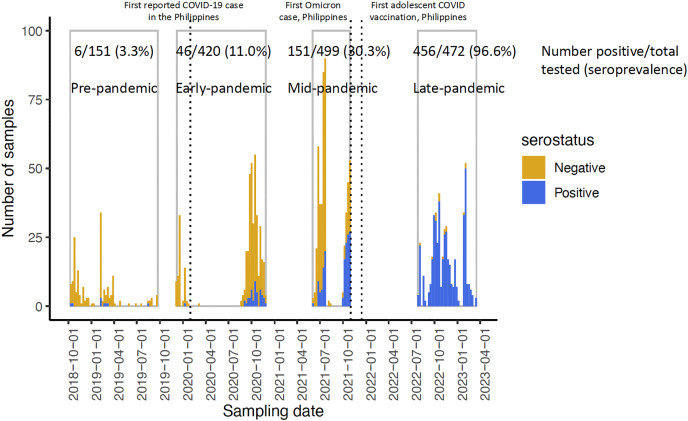
Number and percent seropositive to SARS-COV-2 measured by RBD ELISA in the cohort for samples collected across four timepoints over the pandemic.

Samples were grouped by cohort sampling timepoint: pre-pandemic (October 2018 to September 2019), early in the pandemic (timepoint 2, December 2019 to November 2020; before the emergence of Delta), mid-pandemic (timepoint 3, June to October 2021; during Delta wave and before Omicron surge), and late pandemic (timepoint 4, July 2022 to March 2023); following Omicron emergence and during mass vaccination for adolescents (starting in November 2021) (Fig 3). Seropositivity increased from 11.0% early in the pandemic to 30.3% by mid-pandemic. By the late pandemic sampling, 96.6% of the cohort were seropositive to SARS-CoV-2, following the Omicron wave and adolescent vaccination.

Among participants who were seropositive at early or mid-pandemic timepoints, testing of pre-pandemic samples identified SARS-CoV-2 antibodies in only 6/151 (4.0%). We compared antibodies to the RBDs of the four endemic coronaviruses (OC43, HKU1, 229E, NL63) in these 6 pre-pandemic samples with pre-pandemic samples from 61 other individuals randomly selected from among those with antibodies to SARS-CoV-2 at timepoint 3 and described previously [19]. The responses showed no significant differences for OC43, HKU1, or 229E (Wilcoxon rank-sum test, p > 0.05), although slightly higher NL63 antibodies were observed (p = 0.02).

**Fig 4:**
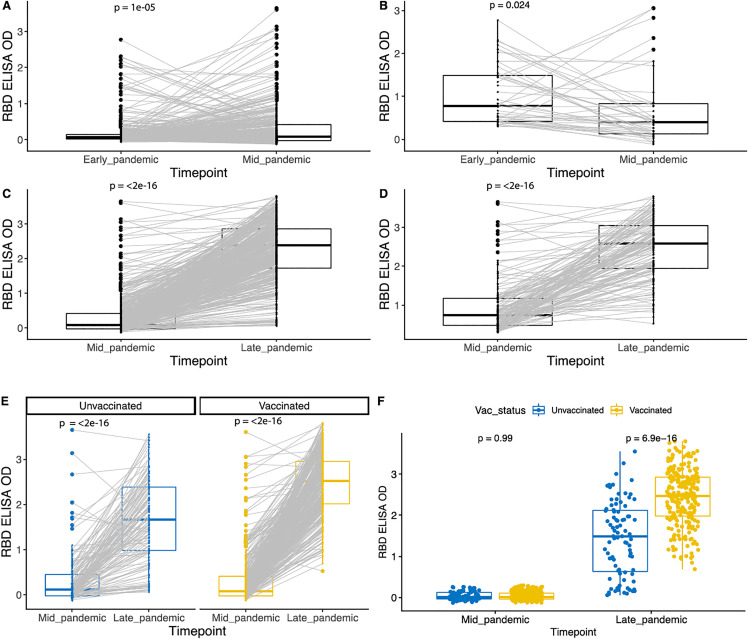
Antibodies to SARS-CoV-2 RBD increased over the course of the pandemic. **(A)** Comparison of ELISA OD values between early- (2019/2020) to mid-pandemic (2021) for all study participants or **(B)** only for individuals who were already seropositive at the pandemic sampling. **(C)** Comparison of ELISA OD values between mid- to late-pandemic for all participants or **(D)** only those who were positive at the mid-pandemic timepoint. **(E)** Comparison of mid- vs. late-pandemic ELISA OD values, separated by vaccination status. Antibody increase mid- to late-pandemic in both vaccinated and unvaccinated individuals. **(F)** Antibody response in seronegative individuals comparing vaccinated and unvaccinated at mid and late pandemic. ELISA OD values between pandemic periods compared with paired t-tests with Bonferroni correction.

We next evaluated how individual RBD antibody OD values shifted from early to mid-pandemic, which corresponded to the period from when the ancestral SARS-CoV-2 was circulating during the Delta wave. Overall, SARS-CoV-2 RBD binding increased significantly from 2019/2020–2021 (p < 0.001) (Fig 4A), with most mid-pandemic seropositive participants having seroconverted during this interval. Interestingly, of the 46 children (11% of the total measured) who were seropositive at the early-pandemic sampling, 19 sero-reverted by mid-pandemic (Fig 4B) and 34 (74% of those positive at the early sampling) showed a decline in ELISA OD values (Fig 4B).

We then tested how SARS-CoV-2 antibodies shifted from mid to late pandemic, which corresponded to the emergence of Omicron and mass vaccination of adolescents in the Philippines. Of the 499 participants, 344 (69.1%) had received full COVID-19 vaccination: 314 (91.3%) Pfizer, 16 (4.64%) Moderna, 8 (2.32%) Sinovac, 5 (1.5%) AstraZeneca and 1 (0.3%) Janssen (S2 Table). In addition, 13/499 (2.6%) were partially vaccinated: 12 (92.31%) Pfizer and 1 (7.69%) Moderna. Consistent with high vaccination rates in this population during this period, SARS-COV-2 RBD ELISA OD values increased significantly between mid to late pandemic (p < 0.0001) and a large fraction of children seroconverted for the first time to SARS-CoV-2 (66%) (Fig 4C).

Of the remaining children, 29% were already seropositive by mid-pandemic, and this group overall maintained or had an increase in OD values (p < 0.0001, Fig 4D) with only 2% experiencing a decline. Additionally, a small fraction remained seronegative (3%).

To determine how much the increase in seropositivity was due to vaccination or also to natural infection, we evaluated change in antibodies from mid to late pandemic stratified by those who did versus did not receive the vaccine (Fig 4E). Antibodies increased most in those who were vaccinated but also increased significantly in those who remained unvaccinated (p < 0.001). When we compared those who seroconverted for the first time between mid to late pandemic to evaluate whether natural infection alone induced similar OD values to those with vaccination (with or without additional natural infection), we found the vaccinated group had higher OD values compared to the unvaccinated group (p < 0.0001, Fig 4F).

**Fig 5:**
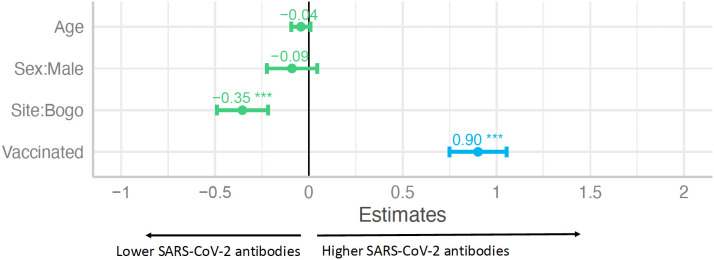
Vaccination was associated with increased magnitude while Bogo site associated with decreased magnitude of SARS-CoV-2 RBD antibodies. The linear model was adjusted for vaccine status, site, sex, and age. *** indicates p value<0.001.

Finally, we evaluated what factors were most strongly associated to seropositivity to SARS-CoV-2 measured in the late pandemic samples. We performed a multivariable linear regression and found that vaccination was the strongest predictor of ELISA OD, associated with an increase of 0.9 in OD values compared to unvaccinated (p < 0.001), while living in Bogo was associated with lower OD values (0.35 OD lower than those in Balamban, p < 0.001) (Fig 5). This observation was interesting because Balamban had a lower vaccination rate than Bogo, indicating differences in SARS-CoV-2 positivity by site.

## Discussion

Our study describes how antibodies to SARS-CoV-2 changed in an adolescent population in Cebu, Philippines over the course of the COVID-19 pandemic, both due to natural infection and vaccination. By leveraging a well-established population-based dengue cohort, we were able to use archived pre-pandemic samples and conduct longitudinal follow-up across multiple pandemic phases. This approach provides insight into shifts in SARS-CoV-2 seroprevalence in an understudied region.

Despite more than four million reported COVID-19 cases nationally, relatively few infections were reported among individuals younger than 19 years of age in 2021 [20]. In contrast, we found that 30.3% of adolescents aged 9–14 years old were already positive for SARS-CoV-2 antibodies in the same year, indicating substantial under-ascertainment of infection in this age group. To our knowledge, this is the first study that reported SARS-CoV-2 seroprevalence among children in a semi-urban community setting in the Philippines over the course of the COVID-19 pandemic.

Previous SARS-CoV-2 seroprevalence studies in the Philippines were largely conducted in tertiary hospitals in Manila among patients/household contacts and healthcare workers reporting seroprevalences ranging from 11.3% to 46.8% [7] and 28.8% to 65.1% [6]. Malijan, et al., reported higher (42.1%) SARS-CoV-2 seroprevalence among children less than 18 years old who sought consult in a hospital in Manila. The difference in our reported seroprevalence may be due to the age group restriction of our study participants to 9–14 years. Nevertheless, our report also included measurement of SARS-CoV-2 seropositivity after the COVID-19 vaccination roll out among adolescents in October 2021 and found that 96.6% of children were SARS-CoV-2 seropositive (100% among vaccinated, 88% among unvaccinated) by 2022–2023.

A small fraction of adolescents (3%) remained seronegative, likely because some participants were unvaccinated or only partially vaccinated against COVID-19. In addition, some may have mounted less durable antibody responses, with antibody levels waning below the ELISA detection threshold by the time of sampling.

We detected anti-SARS-CoV-2 antibodies in a small number [6] of samples prior to the first reported case in the country. We did not observe a significant difference in ELISA OD values to the endemic coronaviruses for these participants compared to others in the cohort who were SARS-CoV-2 negative, but we cannot rule-out cross-reactions due to more recent coronavirus infections, which have been shown in previous studies to result in cross-reactivity to SARS-CoV-2 [21–28].

The change in RBD binding antibodies to SARS-CoV-2 between the early to mid-pandemic timepoint suggests there may have been antibody waning for those who were not re-exposed. Previous studies in pediatric populations have measured SARS-CoV-2-specific antibodies following acute infections. Dowell and colleagues tested antibody responses 6-months and 12-months post infection in children (3–11 years of age), noting moderate decreases in antibodies levels for spike, RBD, and a significant reduction in nucleocapsid-specific antibodies [29]. Another study by Gentles, et al., showed a significant decrease in anti-nucleocapsid neutralizing antibodies at 24–29 weeks as compared to those at 8–13 weeks post symptom onset in children (<18 years old) [30]. Finally, a study by Weisberg et al. found that children with SAR-CoV-2 infection had responses primarily to the spike protein and less neutralizing activity than adults, who had responses to multiple antigens and greater breadth and neutralizing activity [31].

We observed that antibodies increased due to vaccination and natural infection between mid to late pandemic sampling, with differences in transmission intensity by site evident in ELISA values. This observation suggests a portion of the increase in ELISA OD values was due to vaccination, but a large fraction was also due to natural infection during this period, likely because of the emergence of Omicron. Differences in antibody magnitude by site suggest higher transmission intensity in Balamban, likely due to its proximity to highly urbanized areas and greater population movement, compared with Bogo City, which is more geographically isolated location in the northern part of the island.

Despite the low RT-PCR positivity, there was a high level of SARS-CoV-2 seropositivity, even prior to COVID-19 vaccine roll-out, reflecting limited acceptance of diagnostic testing. Reluctance to test was driven by fear of nasopharyngeal swabs, concern about testing positive, and avoidance of quarantine during periods of strict mobility restrictions. Discomfort and psychological stress associated with swab-based testing in children, limited early testing capacity and evolving eligibility guidelines likely further reduced participation. In addition, stringent quarantine policies, requiring isolation of cases and household contacts until clearance by local health authorities, may have discouraged testing [32–35]. Less invasive sampling methods (use of saliva samples), enhanced risk communication (including demonstration videos of procedure) and closer coordination with local health authorities may improve testing acceptability in future surveillance efforts [36,37].

The study is limited to adolescents enrolled in a pre-existing dengue cohort (aged 11–14 years at time of enrolment) and does not represent younger children or older age groups. Data were collected from two semi-urban sites in Cebu, which limits national generalizability but does not affect inference within this population. Although uptake of RT-PCR testing for febrile illness was low, the primary outcome relied on serologic measures rather than case detection. Furthermore, only 22% of 134 reported febrile episodes provided consent and there may be significant differences between the children who consented to participate in the study and those who declined, particularly in terms of health-seeking behavior, socio-economic status, and other relevant factors. Nonetheless, the longitudinal design, access to archived samples collected over time and statistically sufficient sample size provide a unique opportunity to assess antibody dynamics that cannot be replicated in cross-sectional studies.

This prospective cohort in an understudied adolescent population provides insight into SARS-CoV-2 seroprevalence and antibody dynamics over the course of the pandemic. We document early circulation, with 30.3% SARS-CoV-2 seropositivity by 2021, and demonstrate how immunity accumulated through both vaccination and natural infection, reaching 96.6% seropositivity during the late pandemic following the emergence of Omicron. These findings complement predominantly cross-sectional and hospital-based seroprevalence studies by providing longitudinal, community-based evidence from a low- and middle-income country setting, where pediatric data remain limited. Beyond COVID-19, this study highlights the public health value of leveraging existing disease-specific cohorts for rapid, cost-effective surveillance of emerging pathogens and more accurate estimation of infection burden in settings with limited routine case reporting. Although the COVID-19 pandemic has ended, continued circulation of SARS-CoV-2 and reports of resurgences in Asia underscore the importance of sustained monitoring and improved understanding of transmission dynamics in younger populations [38–40].

## Supporting information

S1 Table Characteristics and outcomes for participants with confirmed COVID-19 cases. (DOCX)

S2 Table Characteristics of participants based on vaccination status. (DOCX)
