# Supplementary material for: Development of seropositivity to SARS-CoV-2 over the course of the COVID-19 pandemic in adolescents in a longitudinal cohort study in Cebu, Philippines
Source: PLOS Glob Public Health. 2026 Feb 26;6(2):e0005961. doi: 10.1371/journal.pgph.0005961 (PMC12944793; doi:10.1371/journal.pgph.0005961)
Supplement: S1 Table — (DOCX) [file pgph.0005961.s001.docx]

**S1 Table. Characteristics and outcomes for participants with confirmed COVID-19 cases.**

|  | Sex | Site | Age | 2nd vaccine date | COVID test date | Fever duration (days) | Symptoms and duration (days) | Symptoms duration (days) |
| --- | --- | --- | --- | --- | --- | --- | --- | --- |
| DS1960 | F | Balamban | 14 | 1/28/22 | 10/11/22 | 1 | Congestion (3), sore throat (3) | 3 |
| DS0230 | F | Bogo | 17 | 12/21/21 | 1/18/22 | 2 | Cough (4), anosmia & augesia (4) | 4 |
| DS0483 | M | Bogo | 14 | Unvaccinated | 1/20/22 | 2 | Cough (3), congestion (2) | 3 |
| DS2705 | F | Bogo | 15 | 12/20/21 | 7/29/21 | 2 | Cough (3), headache (1) | 3 |
| DS2825 | F | Bogo | 14 | 12/20/21 | 1/19/22 | No fever | N/A | N/A |
