# Supplementary material for: Development of seropositivity to SARS-CoV-2 over the course of the COVID-19 pandemic in adolescents in a longitudinal cohort study in Cebu, Philippines
Source: PLOS Glob Public Health. 2026 Feb 26;6(2):e0005961. doi: 10.1371/journal.pgph.0005961 (PMC12944793; doi:10.1371/journal.pgph.0005961)
Supplement: S2 Table — (DOCX) [file pgph.0005961.s002.docx]

**S2 Table. Characteristics of participants based on vaccination status.**

| Characteristic | Full^1^, N=344 | Partial^1^, N=13 | Unvaccinated^1^, N=142 |
| --- | --- | --- | --- |
| Site  Balamban  Bogo | 179 (52%)  165 (48%) | 7 (54%)  6 (46%) | 99 (70%)  43 (30%) |
| Sex  Female  Male | 205 (60%)  139 (40%) | 3 (23%)  10 (77%) | 65 (46%)  77 (54%) |
| Brand  Astra Zeneca  Janssen  Moderna  Pfizer  Sinovac  Unvaccinated | 5 (1.5%)  1 (0.3%)  16 (4.7%)  314 (91%)  8 (2.3%)  0 (0%) | 0 (0%)  0 (0%)  1 (7.7%)  12 (92%)  0 (0%)  0 (0%) | 0 (0%)  0 (0%)  0 (0%)  0 (0%)  142 (100%) |
| ^1^ n (%) | | | |
